# Subcutaneous Adipose Tissue Radiation Attenuation Is Associated With Increased 1‐Year Mortality in Polytrauma Patients

**DOI:** 10.1002/jcsm.13743

**Published:** 2025-10-13

**Authors:** Leanne L. G. C. Ackermans, Jasper C. Stokroos, David P. J. Van Dijk, Bjorn Winkens, Martijn Poeze, Leonard Wee, Ralph Brecheisen, Steven M. W. Olde Damink, Jan A. Ten Bosch, Taco J. Blokhuis

**Affiliations:** ^1^ Department of Traumatology Maastricht University Medical Centre+ Maastricht The Netherlands; ^2^ Department of Surgery, NUTRIM School of Nutrition and Translational Research in Metabolism Maastricht University Medical Centre+ Maastricht The Netherlands; ^3^ Department of Surgery Maastricht University Medical Centre+ Maastricht The Netherlands; ^4^ Department of Methodology and Statistics, Faculty of Health, Medicine and Life Sciences Maastricht University Maastricht; ^5^ CAPHRI Care & Public Health Research Institute Maastricht University Medical Centre+ Maastricht The Netherlands; ^6^ Department of Radiation Oncology (MAASTRO), GROW School for Oncology and Development Biology Maastricht University Medical Centre+ Maastricht The Netherlands; ^7^ Clinical Data Science, Faculty of Health Medicine and Lifesciences Maastricht University Maastricht The Netherlands; ^8^ Department of General, Visceral and Transplantation Surgery RWTH University Hospital Aachen Aachen Germany

**Keywords:** adipose tissue, body composition, computed tomography, mortality, polytrauma

## Abstract

**Background:**

Polytrauma patients with an Injury Severity Score (ISS) ≥ 16 have a high mortality rate. Early identification of patients at risk of mortality is key. Different risk stratification models are available; however, body composition on third lumbar computed tomography (L3 CT) is not routinely used. The aim of this study is to determine the effect of CT body composition on 1‐year mortality in adult polytrauma patients.

**Methods:**

Body composition analysis (L3 CT) was performed on 593 adult polytrauma patients. The associations with 1‐year mortality were assessed using uni‐ and multivariable logistic regression analysis. As a sensitivity analysis, 1‐year mortality was analysed using Kaplan–Meier survival curves, log‐rank tests and Cox regression.

**Results:**

The study population was predominantly male (69.5%), with a mean age of 55 (±20) years and an average BMI of 25.34 kg/m^2^ (±4.07). Comorbidities were present in 327 (55.4%) patients, with an average Charlson Comorbidity Index (CCI) of 2.07 points (±2.1). The mean ISS score was 27.59 (±11.06); 323 (54.5%) patients had an ISS ≥ 25 points. Age, CCI, ISS, skeletal muscle index and skeletal muscle radiation attenuation (OR 1.053, 5.713, 3.711, 0. 563 and 0.533, respectively; *p* < 0.001), subcutaneous adipose tissue radiation attenuation (SATRA OR 1.253, *p* = 0.028) and visceral adipose tissue index (OR 1.242, *p* = 0.038) were significantly associated with 1‐year mortality. In multivariable logistic regression, age, ISS and SATRA remained statistically significantly associated with 1‐year mortality (OR 1.062, *p* < 0.001; OR 4.761, *p* < 0.001; OR 1.396, *p* = 0.009).

**Conclusions:**

This study demonstrated that subcutaneous adipose tissue radiation attenuation on emergency trauma CT scans is significantly associated with 1‐year mortality in adult polytrauma patients. Additionally, we found a significant effect of age and ISS on 1‐year mortality. Incorporating body composition analysis could lead to a better selection of patients at risk for 1‐year mortality and aid in treatment decision‐making.

## 1 Introduction

Every year approximately 75 000 trauma patients are admitted to Dutch hospitals [1]. Up to 7% of these patients are defined as polytrauma patients, with an Injury Severity Score (ISS) greater than or equal to 16 [2]. Polytrauma is a life‐changing event with a large burden of disease. Moreover, mortality after trauma is high. In 2021, the Dutch in‐hospital mortality rate for polytrauma patients reached up to 18% [2]. Therefore, early identification and prediction of patients with an increased risk of mortality is important.

Currently, several methods can be used to predict survival in trauma patients [3]. The most widely used are the ISS [4] and the Trauma and Injury Severity Score (TRISS) [5]. These scoring systems use different parameters, such as patient characteristics, physiological status and mechanism of trauma. However, body composition is rarely taken into account in mortality risk assessments, though it is proven to be useful in different patient populations [6]. Previous studies stated that clinical conditions influencing body composition, such as obesity, sarcopenia and cachexia, could have an impact on mortality and morbidity [7, 8].

Body composition can be measured in several ways. The most common measurement of body composition is body mass index (BMI), which is an anthropometric tool defined as the weight in kilograms divided by the square of height in metre [9]. Even though it is used often, BMI has limitations [9]. It does not determine distribution and quality of skeletal muscle or adipose tissue. Additionally, the documentation of BMI is often inadequate in trauma patients [9, 10]. A more specific method for body composition quantification is segmentation on computed tomography (CT) images at the level of the third lumbar vertebra. This is a representation of objective body composition, providing not only both the area and quality of skeletal muscle tissue but also subcutaneous and visceral adipose tissue [11].

CT body composition analysis on the third lumbar level is a well‐known assessment in conditions such as sarcopenia, obesity and cachexia [6, 12]. At this level, the surface area of skeletal muscle, subcutaneous and intra‐abdominal adipose tissues are valid representations of the total body volumes [13, 14]. Radiation attenuation indicates how much radiation is absorbed in the body tissues. In previous studies, skeletal muscle index (SMI), low skeletal muscle radiation attenuation (SMRA) and high visceral adipose tissue (VAT) were all associated with a poor clinical outcome [15, 16]. With the broad availability of CT scans in polytrauma patients, valuable information on body composition could be easily extracted, providing an inexpensive and efficient instrument for body composition quantification [17].

Although CT scans are obtained routinely in polytrauma patients, only a few studies show the clinical relevance of body composition on CT and outcome for this patient group. Kaplan et al. showed prediction of 1‐year mortality in trauma patients with sarcopenia and osteopenia [18]. Other studies show associations between body composition and clinical outcome in trauma patients. However, these studies have limitations such as small sample sizes, different outcome measurements and cut‐off values for sarcopenia. Moreover, some studies only measured skeletal muscle area, but adipose tissue area or radiation attenuation was not measured [18, 19, 20, 21]. Because of the large availability of CT images in polytrauma patients, a rigorous assessment of the association between body composition measurements and clinical outcome could have a substantial impact in daily practice.

The aim of this study is therefore to determine the effect of body composition on 1‐year mortality and in adult polytrauma patients.

## 2 Methods

### 2.1 Study Design

This is a retrospective observational cohort study of polytrauma patients in a single Level I trauma centre. Patient records of the Trauma Registry retrieved from the database of the regional acute care network (Netwerk Acute Zorg Limburg) were analysed. This database is a comprehensive registration of all trauma patients who are admitted to the hospital. Approval for this study was supplied by the local ethics committee of the Maastricht University Medical Centre (MUMC‐2018‐0756). Requirement for informed consent was waived because of the retrospective nature of this study.

### 2.2 Patients and Data Collection

Data from adult polytrauma patients, defined as an ISS ≥ 16, who were admitted between January 2015 and December 2021 were assessed for study eligibility. A CT scan of sufficient image quality, meaning no radiation artefacts and low dose, at the level of L3 obtained within 24 h upon arrival had to be available for image processing. Moreover, patients' height and mortality data had to be available. Lastly, data from patients who died within 24 h upon admission due to severe neurologic trauma were excluded. Baseline patient characteristics were extracted from the electronic records by two observers (L.L.G.C.A. and J.S.) The characteristics included sex, age, body height, body weight, BMI, Charlson Comorbidity Index (CCI, divided into three groups: 0, 1–2 and 3+). The data were based on a prospective registration of all admitted trauma patients. In this database, registration of the used parameters is mandatory, thereby minimizing the likelihood of missing data. A list of abbreviations can be found in Table S1.

### 2.3 Body Composition Analysis

CT‐scan images on third lumbar level within 24 h on arrival were selected by a single researcher (L.L.G.C.A.) trained in body composition analysis, according to the guidelines of the Alberta Protocol [22]. Body composition parameters of skeletal muscle tissue (SM), visceral adipose tissue (VAT) and subcutaneous adipose tissue (SAT) were measured, and cross‐sectional surface areas (cm^2^) were calculated using a validated deep learning neural network (MosaMatic, MUMC+, The Netherlands) [23] and visually checked afterwards. The HU threshold for adipose tissue was defined between −190 and −30 HU and for skeletal muscle between −29 and 150 HU. All body composition areas (SM, VAT and SAT) were corrected for patient height and are presented as indices (cm^2^/m^2^). The mean radiation attenuation (RA) was calculated for each patient, and SM, VAT and SAT are presented as SMRA, VATRA and SATRA. The RA, expressed in Hounsfield units (HU), indicates the mean radio density in the body composition parameter.

To correct for the effect of sex on the body composition parameters, Z‐scores were calculated. The Z‐score of each body composition parameter depicts how many standard deviations a patients' score differs from the mean value of the same sex. It was calculated by subtracting the sex specific mean from its value and dividing this difference by the sex specific standard deviation.

### 2.4 Outcome Measures

The primary outcome measure was all‐cause 1‐year mortality. The secondary outcome was overall survival. Mortality data were obtained by two researchers (L.L.G.C.A. and J.A.S.) at January 2023 and censored at 1 year from the admission arrival date.

### 2.5 Statistical Analysis

Numerical data are presented as mean with standard deviation (SD), whereas frequencies and percentages were used for categorical data. Patient characteristics were assessed by either a χ2 test or an independent‐samples *t*‐test. To identify relevant baseline characteristics with a potential relation to 1‐year mortality, we first performed univariable logistic regression analyses. Additionally, all variables were included in the multivariable logistic regression analysis, including the Z‐scores of body composition parameters (SMI, SMRA, VATI, VATRA, SATI and SATRA) as independent variables, after which the backward stepwise elimination procedure was applied to see which variables remained significant. As a sensitivity analysis, the associations with 1‐year mortality were also analysed using survival analysis, that is, Kaplan–Meier survival curves, log‐rank tests and multivariable Cox regression analysis. Thresholds for body composition analysis are presented if statistically significant in the multivariable model. These thresholds were calculated using sex‐specific tertiles in line with other studies, based on previously validated methods [24]. Two‐sided *p* values ≤ 0.05 were considered statistically significant. All statistical analyses were performed using IBM SPSS Statistics for Windows (Version 27.0; Armonk, NY, USA).

## 3 Results

### 3.1 Patient Characteristics

**FIGURE 1:**
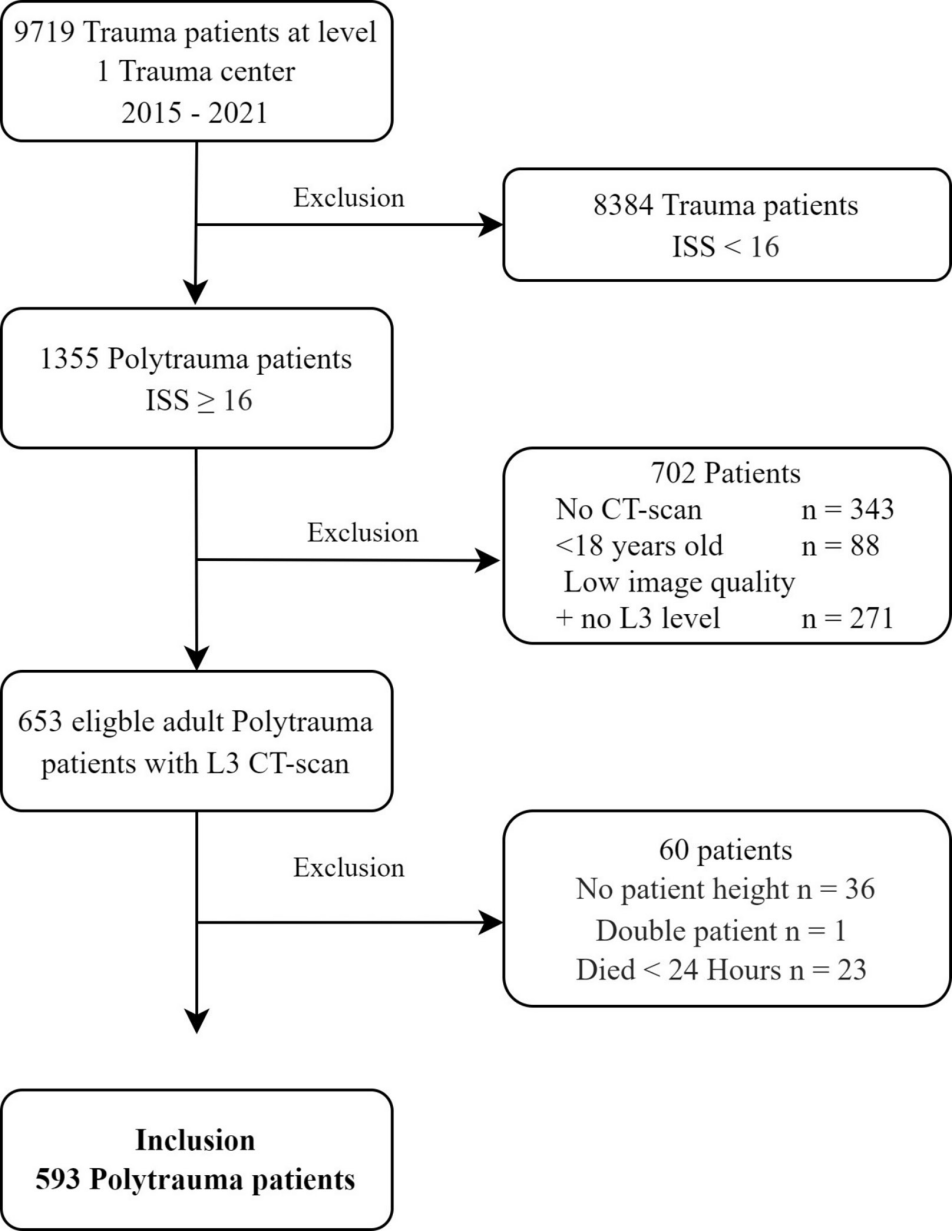
Flowchart of patient selection and exclusion.

From 2015 to 2021, 9719 trauma patients presented at the emergency department of the Level I trauma centre. Of these, 1335 had an ISS ≥ 16. In total, data from 762 patients (13%) were discarded due to missing CT images, poor CT image quality or missing patient parameters such as height or loss to follow‐up. Patients who died within 24 h after arrival in the emergency department due to severe neurologic trauma, and their data were excluded from analysis. One patient presented at the emergency department multiple times and was only included for the statistical analysis during the first visit. In total 593 patients were included. A flowchart of patient selection has been included as Figure 1.

Baseline patient characteristics are displayed in Table 1. The study population was predominantly male (69.5%), with a mean age of 55 (±20) years and an average BMI of 25.34 kg/m^2^ (±4.07). Comorbidities were present in 327 (55.4%) patients, with an average CCI of 2.07 points (±2.1). The mean ISS score was 27.59 (±11.06); 323 (54.5%) patients had an ISS ≥ 25 points.

**TABLE 1:** Patient characteristics.

	Total study population	
Number of patients	593	100%
Sex		
Males	412	69.5%
Females	181	30.25%
Age (years)		
18–30	102	17.2%
30–40	56	9.4%
40–50	53	8.9%
50–60	122	20.6%
60–70	106	17.9%
70–80	98	16.5%
80+	56	9.4%
BMI (kg/m^2^)		
Underweight	13	2.2%
Normal weight	299	50.4%
Overweight	212	35.8%
Obese	56	9.4%
Morbid obese	13	2.2%
CCI		
0	194	32.7%
1–2	178	30.1%
3–4	128	21.6%
≥ 5	93	15.6%
ISS		
16–24	270	45.5%
≥ 25	323	55.5%
Mortality		
Overall^a^	120	20.2%
< 1 month	86	14.0%
< 1 year	102	17.2%

^a^ Observed in January 2023, 18 patients died after 1 year follow‐up; these patients were censored at 1‐year follow‐up in the analysis of 1‐year mortality.

### 3.2 Body Composition Analysis

**FIGURE 2:**
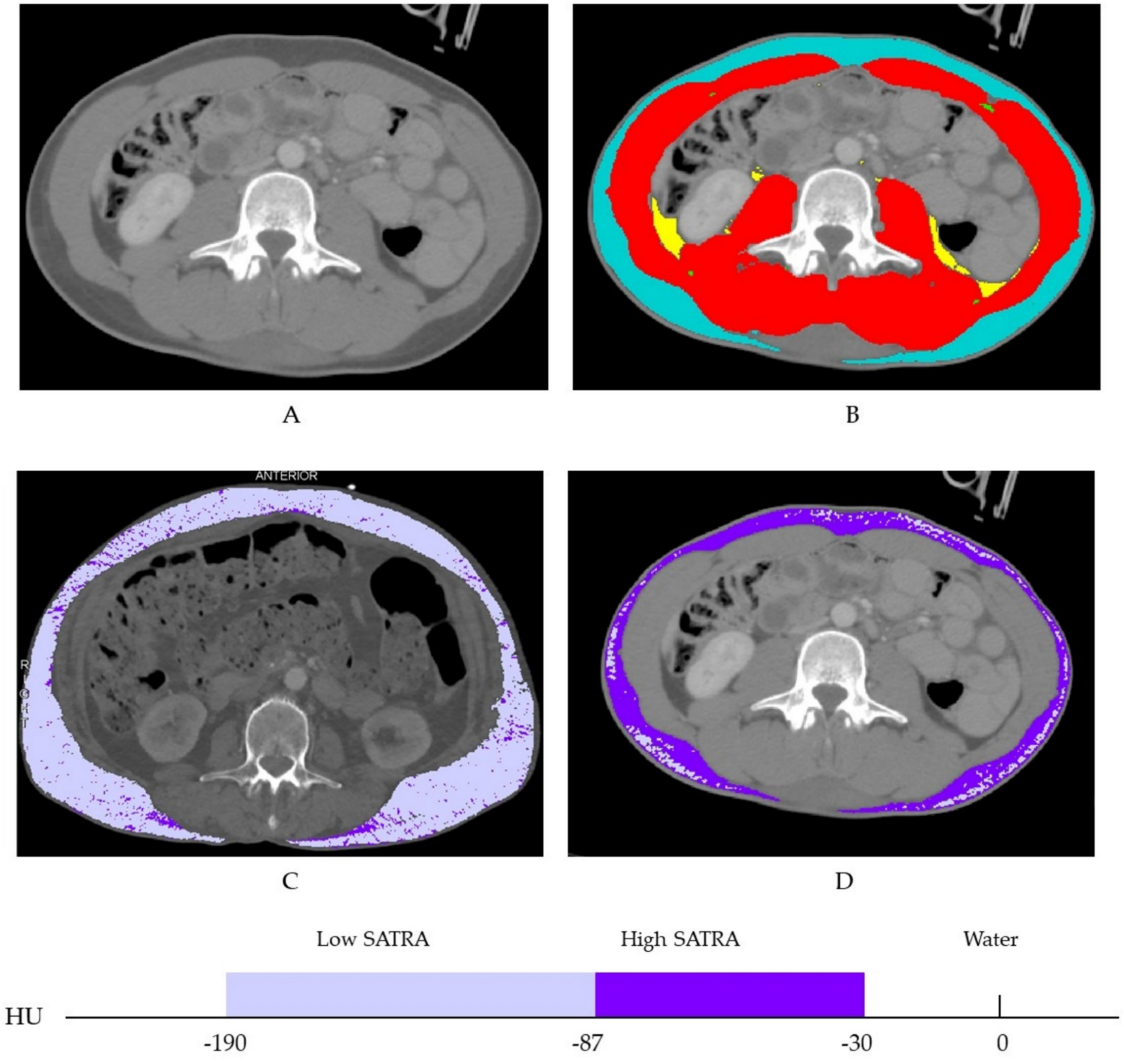
Body composition analysis at the third lumbar vertebra with SATRA characteristics of two patients applied for SATRA assessment. (A) Original CT slice on third lumbar level. (B) Segmented skeletal muscle, visceral adipose tissue and subcutaneous adipose tissue are delineated in red, yellow and blue, respectively. (C,D) Comparison of two patients with (C) a high low > − 87.54 SATRA and (D) with a low high <−87.54 SATRA (darker shade of purple, indicative of high triglyceride concentration).

Body composition segmentation was performed in all 593 patients. Overall cohort mean and standard deviation are presented in Table 2. In this cohort, males were more muscular (*p* = 0.001) and had more visceral adipose tissue (*p* < 0.001). Females had more subcutaneous adipose tissue (*p* < 0.001). An example of a CT body composition analysis by segmentation on the third lumbar slice can be seen in Figure 2. This figure also highlights the SATRA estimation from two patients in the high and low SAT group. Figure 2C presents a patient with a high SATRA on average (−110 HU). Figure 2D presents a patient with a low SATRA on average (−64 HU).

**TABLE 2:** Mean and standard deviation of six body composition parameters measured on L3 CT, divided for males and females.

	Overall (*n* = 593)	Male (*n* = 412)	Female (*n* = 181)	*p*
	Mean [SD]	Mean [SD]	Mean [SD]	
Skeletal muscle index	49.90 [11.21]	53.90 [10.03]	40.77 [7.98]	0.001
Skeletal muscle radiation attenuation	39.94 [11.35]	42.19 [10.42]	34.80 [11.73]	0.068
Visceral adipose tissue index	41.10 [31.71]	44.97 [33.11]	32.28 [26.34]	< 0.001
Visceral adipose tissue radiation attenuation	−88.38 [9.12]	−89.07 [9.21]	−86.80 [8.73]	0.474
Subcutaneous adipose tissue index	54.04 [33.67]	44.15 [25.88]	76.57 [38.29]	< 0.001
Subcutaneous adipose tissue radiation attenuation	−91.67 [11.59]	−89.84 [11.26]	−95.83 [11.28]	0.516

### 3.3 Logistic Regression Analysis: 1‐Year Mortality

Several parameters were identified in the univariable analysis as significantly associated with 1‐year mortality; age, CCI, ISS, skeletal muscle index, skeletal muscle radiation attenuation (OR 1.053, OR 5.713, OR 3.711, OR 0. 563, OR 0.533, respectively; *p* < 0.001 for all). In addition, visceral adipose tissue index and subcutaneous radiation attenuation showed a significant effect as well (SATRA OR 1.253, *p* = 0.028; VATI OR 1.242, *p* = 0.038). After backward stepwise elimination, only age, ISS and subcutaneous adipose tissue radiation attenuation remained significantly associated with 1‐year mortality (age OR 1.047, *p* < 0.001; ISS ≥ 25 OR 4.751 *p* < 0.001; SATRA OR 1. 396, *p* = 0.009). This is elaborated in Table 3.

**TABLE 3:** Univariable and backward multivariable logistic regression analysis.

	Univariable			Multivariable^a^		
	OR	[95% CI]	*p*	OR	[95% CI]	*p*
Sex						
Male	Ref	—	—			
Female	1.37	[0.88–2.15]	0.167	—	—	—
Age	1.05	[1.04–1.07]	< 0.001	1.05	[1.02–1.07]	< 0.001
Charlson Comorbidity Index						
0–2	Ref	—	—			
≥ 3	5.71	[3.56–9.17]	< 0.001	1.90	[0.89–4.09]	0.099
Injury Severity Score						
16–24	Ref	—	—			
≥ 25	3.71	[2.24–6.14]	< 0.001	4.75	[2.74–8.23]	< 0.001
Emergency surgery	1.24	[0.76–2.00]	0.387	—	—	—
Muscle Area Index*	0.56	[0.52–0.82]	< 0.001	—	—	—
SAT Area Index*	1.07	[0.87–1.32]	0.511	—	—	—
VAT Area Index*	1.24	[1.01–1.52]	0.038	—	—	—
Muscle Radiation Attenuation*	0.53	[0.43–0.67]	< 0.001	—	—	—
SAT Radiation Attenuation*	1.25	[1.02–1.53]	0.028	1.40	[1.09–1.80]	0.009
VAT Radiation Attenuation*	1.13	[0.92–1.40]	0.252	—	—	—

^a^ Variable(s) entered in starting model of the backward stepwise elimination procedure: Sex, Age, Charlson Comorbidity Index, Injury Severity Score, Emergency surgery, Muscle Area Index*, SAT area Index*, VAT Area Index*, Muscle Radiation Attenuation*, SAT Radiation Attenuation*, VAT Radiation Attenuation*. * indicates Z‐scores were used.

### 3.4 Survival Analysis: 1‐Year Mortality

**FIGURE 3:**
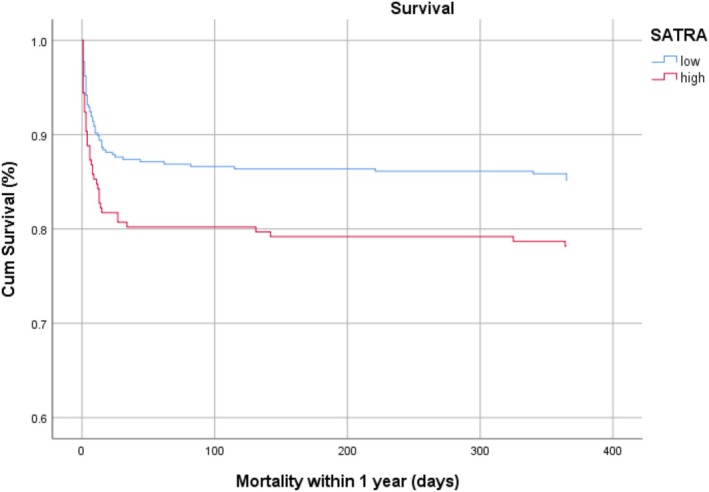
Kaplan–Meier curve for high SATRA (red) versus low SATRA. Threshold were based on the highest tertile (HU for males −87.54, females −93.12).

Overall mortality occurred in 120 patients (20.2%). 1‐Year mortality occurred in 102 (17.2%) patients. Neurologic trauma was the most frequent cause of death (*n* = 54); other causes were cardiac, pulmonary, oncology or unknown. Most of the patients (*n* = 86) died in the first 30 days after arrival, which can be seen in the Kaplan–Meier curves in Figure 3. Survival analysis was performed by dividing SATRA into two groups with the highest tertile as a threshold value. Survival analysis showed similar results as logistic regression analysis. Log‐rank analysis showed that a high SATRA was significantly associated with shorter overall survival (*p* = 0.030). Cox regression analysis revealed SATRA remained significant after adding age, CCI and ISS to the model. Hazard Ratios can be found in Table S2.

When using threshold values to define sarcopenia (SMI < 48.95 cm^2^/m^2^ for males and SMI 36.93 cm^2^/m^2^ for females) and myosteatosis (SMRA < 36.88 HU for males and SMRA < 29.25 HU for females), Cox multivariable regression with correction for potential confounders did not show significant effects for these variables.

## 4 Discussion

For adult polytrauma patients with an ISS ≥ 16, a high subcutaneous adipose tissue radiation attenuation on third lumbar CT was significantly associated with 1‐year mortality. Furthermore, older patients or patients with an ISS greater than or equal to 25 had a significantly higher risk of 1‐year mortality. Given the radiologically identified abnormalities in adipose tissue, this study proposes an addition to current risk models for trauma patients. To our knowledge, this is the first large retrospective cohort study to demonstrate an association between subcutaneous adipose tissue radiation attenuation measured on L3 CT and 1‐year mortality in a trauma population. A high SATRA is a novel risk indicator for mortality in these patients.

SATRA as a body composition parameter has never been mentioned as a risk indicator in a trauma population. However, similar findings have been reported an association between high SATRA and mortality in patients with cirrhosis [25] as well as patient with multiple myeloma [26]. A high SATRA was also similarly associated with postoperative complications in surgery patients [27].

Adipose tissue radiation attenuation measured by CT has been introduced as an indirect marker of tissue quality [28]. Several other factors might impact SATRA, such as blood flow [29], adipocyte size [30] or lipid content [31]. Subcutaneous adipose tissue plays a central role in lipid storage and energy homeostasis; therefore, HU represents multiple aspects of tissue quality. A high SATRA might indicate exhaustion of reserves or diminished lipid storage capacity, resulting in unfavourable clinical outcomes. A low adipose tissue radiodensity could indicate that the fat contains higher levels of lipid content and could be indicative of better nutritional status [27, 32]. Adipose tissue with higher lipid content and lipolytic activity can increase systemic free fatty acids [31]. Ebadi et al. shows that if a low SATRA is detected, the adipocytes are smaller. This indicates more lipid storage capacity and a higher physiological reserve [25]. They even state that the quality of adipose tissue (SATRA) is an earlier predictor of mortality compared to the quantity of subcutaneous adipose tissue (SATI), because the morphological quality decline in SAT occurs before the adipose tissue loss. Our study results complement these statements.

Adipose tissue remodelling occurs early and might lead to adipose tissue dysfunction [33]. Therefore, body composition analysis at hospital arrival could be beneficial for patient care. Former L3 CT analyses focused on cancer cachexia, physical frailty and sarcopenia and often included only skeletal muscle parameters such as SMI or SMRA [34]. Recent meta‐analyses identified skeletal muscle mass as a prognostic marker for several clinical outcomes in various patient cohorts [35, 36, 37]. However, a more robust approach was applied in the current study, as we did not narrow the population to only elderly [18]. Even so, previous literature used binary cut‐off values for sarcopenia and myosteatosis, with Prado et al. as the most cited article. Additional limiting factors include false positives and negatives resulting from the reference patient population, which consists of obese individuals and are therefore not globally representative and the studies lack of external validation [19, 38, 39, 40]. The current study overcomes these limitations as skeletal muscle and adipose tissue body composition parameters were expressed as Z‐scores on a continuous scale.

The present study showed several risk factors of 1‐year mortality in the univariable analysis. Low SMI and low SMRA did not remain significant in the multivariable analysis. In previous literature [38, 40], sex‐specific thresholds were established in cancer patients to define sarcopenia and body composition [24]. When testing with similar thresholds in the current study, the results did not show significant results.

Neurologic trauma reflects in the mortality rate, whereas the majority of deaths were within the first 30 days after arrival. This is common in a trauma population [2, 3], but interpreting the 1‐year mortality could be of large influence. A large number of patients in our initial population died of neurologic injury within the first 30 days. These deaths are unlikely to have been influenced by overall patient health, and therefore, we excluded patients who died of neurologic trauma within 24 h after arrival.

The current study has limitations that need to be addressed. First, the retrospective nature of this study was a limiting factor as valuable patient information could be lost. Nevertheless, the used patient registry warrants maximum data registration, as this is a mandatory element in the regional trauma organization. Second, this study measured a trauma population with a high injury severity score, and most of these patients died from a neurologic trauma. Neurologic trauma patients will not benefit from body composition analysis. Finally, CT protocols varied between studies, but they need to be consistent to advance the risk stratification in clinical practice.

Based upon the current study results, routine assessment of CT body composition could be implemented in standard trauma care to identify patients at risk. Following severe trauma, abdominal CT scans are routinely obtained in the emergency evaluation upon admission. These CT scans provide accurate information on body composition. Large prospective datasets could provide population reference values using the continuous variables for CT body composition and known risk factors such as age, ISS and CCI. We believe body composition could be used to guide clinical decision‐making for the individual at risk patients.

Although the validity of the data established in this study needs to be determined in larger prospective multicentre studies, to our understanding, this is the largest study so far demonstrating a relation between SATRA and 1‐year mortality in polytrauma patients.

## 5 Conclusion

This large retrospective cohort study demonstrated that subcutaneous radiation attenuation is significantly associated with 1‐year mortality in adult polytrauma patients. Additionally, we found a significant association between 1‐year mortality and ISS and age. Incorporating body composition analysis and timely recognition of patients with a high SATRA may provide the opportunity to develop strategies to maintain SAT quantity and quality. This could prevent further depletion of adipose tissue, which could lead to a better selection of at‐risk polytrauma patients and aid in treatment decision‐making.

## Ethics Statement

Approval for this study was supplied by the local ethics committee of the Maastricht University Medical Centre (MUMC‐2018‐0756). Informed consent was waived due to the retrospective design of this study.

## Conflicts of Interest

The authors declare no conflicts of interest.

## Supporting information


**Table S1.** Abbreviation table.
**Table S2.** Multivariable Cox regression survival model.
