# Supplementary material for: Subcutaneous Adipose Tissue Radiation Attenuation Is Associated With Increased 1‐Year Mortality in Polytrauma Patients
Source: J Cachexia Sarcopenia Muscle. 2025 Oct 13;16(5):e13743. doi: 10.1002/jcsm.13743 (PMC12516153; doi:10.1002/jcsm.13743)
Supplement: Supplementary file 1 — Table S1. Abbreviation table. Table S2. Multivariable Cox regression survival model. [file JCSM-16-e13743-s001.docx]

**Supplemental**

*Supplemental Table 1. Abbreviation table*

| Abbreviations |  | Definition |
| --- | --- | --- |
| SM(i) | Skeletal Muscle (Index) | The total cross-sectional surface area (cm2) of skeletal muscle tissue as measured by segmentation at L3 level on an axial CT-scan. |
| VAT(i) | Visceral adipose tissue (Index) | The total cross-sectional surface area (cm2) of visceral adipose tissue as measured by segmentation at L3 level on an axial CT-scan. |
| SAT(i) | subcutaneous adipose tissue (Index) | The total cross-sectional surface area (cm2) of subcutaneous adipose tissue as measured by segmentation at L3 level on an axial CT-scan. |
| HU | Hounsfield Units | Quantitative scale for describing radio density |
| -RA | Radiation attenuation | Shows the amount of radiation absorbed in the tissue when undergoing a CT-scan. Each type of tissue has different cut-off values. |
| VATRA | Visceral adipose tissue Radiation Attenuation | The amount of radiation absorbed in the visceral adipose tissue when undergoing a CT-scan, measured in Hounsfield Units (HU). |
| SATRA | Subcutaneous adipose tissue Radiation Attenuation | The amount of radiation absorbed in the subcutaneous adipose tissue when undergoing a CT-scan, measured in Hounsfield Units (HU). |
| ISS | Injury Severity Score | A scoring system for assessing trauma severity. It ranges from 1 to 75 and is based on the worst injury of 6 body systems. |
| Index |  | Correction for CT value by dividing by the square root of patients length. |
| Z-score |  | Showing how many standard deviations each patient’s CT value differs from the sex-specific study sample mean. |
| Emergency surgery |  | Classified as lifesaving surgery following damage control principles. Including orthopedic, trauma and neurological emergency surgery. |
| CCI | Charlson Comorbidity Index |  |
| NAZL | Netwerk Acute Zorg Limburg |  |

*Supplemental Table 2. Multivariable Cox regression survival model.*

|  | Multivariable | | |  |  |
| --- | --- | --- | --- | --- | --- |
|  | **HR** | **[95% CI]** | **p- value** |  |  |
|  |  |  |  |  |  |
| Age | 1.04 | [1.02 – 1.06] | <0.001 |  |  |
| Charlson Comorbidity index |  |  |  |  |  |
| 0-2 | Ref | - | - |  |  |
| ≥3 | 1.72 | [0.88 – 3.38] | 0.113 |  |  |
| Injury Severity Score |  |  |  |  |  |
| 16-24 | Ref | - | - |  |  |
| ≥25 | 3.85 | [2.39 – 6.20] | <0.001 |  |  |
| SATRA | 1.31 | [1.07 – 1.59] | 0.007 |  |  |
